# Bioorganometallic derivatives of 4-hydrazino-benzenesulphonamide as carbonic anhydrase inhibitors: synthesis, characterisation and biological evaluation

**DOI:** 10.1080/14756366.2020.1724995

**Published:** 2020-02-10

**Authors:** Jeremie Brichet, Rodrigo Arancibia, Emanuela Berrino, Claudiu T. Supuran

**Affiliations:** a Laboratorio de Química Inorgánica y Organometálica, Departamento de Química Analítica e Inorgánica, Facultad de Ciencias Químicas, Universidad de Concepción, Concepción, Chile; b Dipartimento Neurofarba, Sezione di Scienze Farmaceutiche, Università degli Studi di Firenze, Firenze, Italy

**Keywords:** Bioorganometallic-hydrazones, sulphanilamide, carbonic anhydrase, inhibitors

## Abstract

A series of bio-organometallic-hydrazones of the general formula [{(η^5^-C_5_H_4_)-C(R)=N–N(H)-C_6_H_4_-4-SO_2_NH_2_}]MLn(MLn = Re(CO)_3_, Mn(CO)_3_, FeCp; R=H, CH_3_) were prepared by reaction of formyl/acetyl organometallic precursors with 4-hydrazino-benzenesulphonamide. All compounds were characterized by conventional spectroscopic techniques (infra-red, ^1^H and ^13^C NMR, mass spectrometry and elemental analysis). Biological evaluation as carbonic anhydrase (CA, EC 4.2.1.1) inhibitors agents was carried out using four human/h) isoforms, hCA I, II, IX and XII. The cytosolic isoforms hCA I and II were effectively inhibited by almost all derivatives with inhibition constants of 1.7–22.4 nM. Similar effects were observed for the tumour-associated transmembrane isoform hCA XII (K_I_s of 1.9–24.4 nM). hCA IX was less sensitive to inhibition with these compounds. The presence of bio-organometallic or metallo-carbonyl moieties in the molecules of these CAIs makes them amenable for interesting pharmacologic applications, for example for compounds with CO donating properties.

## 1. Introduction

The carbonic anhydrases (CAs, EC 4.2.1.1) constitute a superfamily of enzymes in all living organism, their essential role being that of catalysing the reversible hydration of CO_2_ to bicarbonate and protons^1–5^. In this way, from two neutral molecules (carbon dioxide and water), a weak base (bicarbonate) and a strong acid (H^+^ ions) are generated with a huge efficacy^5–12^. This is actually the main mechanism used by most living organism for pH regulation^1–4^. CAs are second only to superoxide dismutase for their catalytic efficiency, with some members of the superfamily showing k_cat_/K_M_ values close to the limit of the diffusion-controlled processes, in the range of 10^8^ M^−1^ × s^−1^.^3–8^ The crucial role played by the above mentioned reaction in organisms all over the phylogenetic tree explains why at least eight CA genetic families are known to date: the α-, β-, γ-, δ-, ζ-, η-, θ- and ι-CA classes^4–13^. All of them are metalloenzymes and for a long period it has been considered that zinc is the essential metal ion in all CAs. However, recent studies demonstrated that the γ-CAs are probably Fe(II) enzymes, the ζ-CAs are Cd(II) enzymes and the ι-CAs Mn(II) proteins^2–13^. However, in all of them, a divalent metal hydroxide is the nucleophilic species converting the substrate CO_2_ to bicarbonate in a ping pong type of mechanism. In most CAs, the rate determining step of the catalytic cycle is the proton transfer reaction from a water molecule coordinated to the metal ion to the reaction medium with generation of the nucleophilic enzyme species^2–7^. In α-CAs, this step is generally assisted by a His residue situated in the middle part of the active site, named proton shuttling residue^2–7^, whereas for other CA genetic families the nature and positioning of the proton shuttle residue is less well understood^8–13^.

Inhibition of these enzymes with various classes of CA inhibitors (CAIs) has an extended range of pharmacologic applications, starting with diuretics and antiglaucoma agents^14^, to antiepileptics^15^, antiobesity^16^, or antitumor drugs^17^ and ending with anti-neuropathic pain^18^ anti-ischemia^19^ and anti-arthritis drugs^20^. This is only possible because different hCA isoforms of the 15 known to date are involved in very diverse pathologies. There are currently 5 CA inhibition mechanisms, but detailed structural data is available only for the first four of them^4^^,^^5^. These are (i) zinc binders; (ii) inhibitors which anchor to the zinc coordinated water/hydroxide ion; (iii) inhibitors which occlude the entrance to the active site; (iv) inhibitors which bind out of the active site, and (v) compounds with unknown inhibition mechanism^4^^,^^5^. However, many of the first- and second-generation inhibitors do not show significant isoform selectivity and this is the main reason why such inhibitors used as drugs show a considerable number of side effects^4^^,^^5^^,^^14–16^. For this reason, the drug design of novel types of CAIs is constantly being pursued by many research groups. Here, we report bio-organometallic-hydrazones of the general formula [{(η^5^-C_5_H_4_)-C(R)=N–N(H)-C_6_H_4_-4-SO_2_NH_2_}]MLn(MLn = Re(CO)_3_, Mn(CO)_3_, FeCp; R=H, CH_3_) which were designed in order to obtain organometallic compounds with CA inhibitory properties.

## 2. Experimental

### 2.1. Materials

All manipulations were conducted under an N_2_ atmosphere using Schlenk techniques. The compounds (η^5^-C_5_H_4_CHO)Re(CO)_3_^21^, (η^5^-C_5_H_4_COCH_3_)Re(CO)_3_^22^, (η^5^-C_5_H_4_CHO)Mn(CO)_3_^23^ and 4-hydrazinyl-benzenesulphonamide^24^ were prepared according to published procedures. Ferrocene carboxaldehyde (98%), acetyl ferrocene (95%), acetylcymantrene (98%) and sulphanilamide (99%) were obtained from Sigma-Aldrich (Chicago, IL) and used without additional purification. Solvents such as CH_2_Cl_2_, hexane, acetone, EtOH, DMSO and THF were obtained commercially and purified using standard methods. Infra-red spectra were recorded in solid state (KBr pellet) on a Jasco FT-IR 4600 spectrophotometer. ^1^H NMR spectra were measured on a Bruker spectrometer model ASCEND TM 400 MHz. All NMR spectra are reported in parts per million (ppm, δ) relative to tetramethylsilane (Me_4_Si), with the residual solvent proton resonances used as internal standards. Coupling constants (*J*) are reported in Hertz (Hz), and integrations are reported as number of protons. The following abbreviations were used to describe the peak patterns: s = singlet, d = doublet, t = triplet and m = multiplet. Mass spectra were obtained on a Shimadzu model QP5050A GC-MS at the Laboratorio de Servicios Analíticos, Pontificia Universidad Católica de Valparaíso. Elemental analyses were measured on a Perkin Elmer CHN Analyser 2400.

### 2.2. Synthesis of organometallic-hydrazones [{(η^5^-C_5_H_4_)-C(H)=N–N(H)-C_6_H_4_-4-SO_2_NH_2_}]MLn(1a, 2a, 3a)

To a stirred suspension of 4-hydrazinyl-benzenesulphonamide (1 eq.) in water (12 ml) and three drops of HCl 32%, the formyl-organometallic precursor (1 eq.) was added. The resulting mixture was stirred for 18 h at room temperature. The precipitate obtainedwas washed with water (2 × 10 ml), diethyl ether (2 × 10 ml) and dried in vacuum for 2 h. The hydrazone derivatives (**1a**, **2a**, **3a**) were recrystallized from acetone/hexane (1:5) at −18 °C.

#### 2.2.1. [{(η^5^-C_5_H_4_)-CH=N–N(H)-C_6_H_4_-4-SO_2_NH_2_}]re(CO)_3_ (1a)

This compound was prepared according to the general procedure described above, using in this case: (η^5^-C_5_H_4_CHO)Re(CO)_3_ (91 mg, 0.25 mmol) and 4-hydrazinyl-benzenesulphonamide (47 mg, 0.25 mmol). Brown-yellow solid, yield 48% (64 mg, 0.12 mmol). IR (KBr, cm^−1^): 3373–3262 (νNH/NH_2_); 2022 (νRe-CO); 1922 (νRe-CO); 1590 (νC=N); 1323 (νS-O); 1152 (νS-O). ^1^H NMR (acetone-d_6_): δ 5.65 (t, 2H, *J* = 2.0 Hz, C_5_H_4_); 6.10 (t, 2H, *J* = 2.0 Hz, C_5_H_4_); 6.32 (s, 2H, NH_2_); 7.15 (d, 2H, *J* = 8.8 Hz, C_6_H_4_); 7.69 (d, 2H, *J* = 8.8 Hz, C_6_H_4_); 7.72 (s, 1H, CH=N); 9.89 (s, 1H, NH). ^13^C NMR (acetone-d_6_): *δ* 83.6 (C_5_H_4_); 84.9 (C_5_H_4_); 102.6 (C_5_H_4ipso_); 111.6 (C_6_H_4_); 127.8 (C_6_H_4_); 131.4 (CH=N); 134.4 (C_6_H_4_); 147.7 (C_6_H_4_); 194.4 (Re-CO). Mass spectrum (based on ^187^Re) (*m/z*): 533 [M^+^]; 449 [M^+^ – 3CO]. Anal. (%) Calc. for C_15_H_12_N_3_O_5_SRe: C, 33.83; H, 2.27 and N, 7.89; found: C, 33.63; H, 2.27 and N, 7.78.

#### 2.2.2. [{(η^5^-C_5_H_4_)-CH=N–N(H)-C_6_H_4_-4-SO_2_NH_2_}]Mn(CO)_3_ (2a)

This compound was prepared according to the general procedure described above, using in this case: (η^5^-C_5_H_4_CHO)Mn(CO)_3_ (58 mg, 0.25 mmol) and 4-hydrazinyl-benzenesulphonamide (47 mg, 0.25 mmol). Brown solid, yield 52% (52 mg, 0.13 mmol). IR (KBr, cm^−1^): 3365–3250 (νNH/NH_2_); 2020 (νMn-CO); 1931 (νMn-CO); 1597 (νC=N); 1325 (νS-O); 1147 (νS-O). ^1^H NMR (acetone-d_6_): *δ* 5.05 (t, 2H, *J* = 2.0 Hz, C_5_H_4_); 5.50 (t, 2H, *J* = 2.0 Hz, C_5_H_4_); 6.31 (s, 2H, NH_2_); 7.18 (d, 2H, *J* = 8.8 Hz, C_6_H_4_); 7.64 (s, 1H, CH=N); 7.74 (d, 2H, *J* = 8.8 Hz, C_6_H_4_); 9.90 (s, 1H, NH). ^13^C NMR (acetone-d_6_): *δ* 82.5(C_5_H_4_); 83.0 (C_5_H_4_); 98.3 (C_5_H_4ipso_); 111.6 (C_6_H_4_); 127.8 (C_6_H_4_); 132.5 (CH=N); 134.4 (C_6_H_4_); 148.2 (C_6_H_4_); Mn–CO (not observed). Mass spectrum (*m/z*): 401 [M^+^]; 317 [M^+^ – 3CO]. Anal. (%) Calc. for C_15_H_12_N_3_O_5_SMn: C, 44.90; H, 3.01 and N, 10.47; found: C, 44.09; H, 3.27 and N, 10.66.

#### 2.2.3. [{(η^5^-C_5_H_4_)-CH=N–N(H)-C_6_H_4_-4-SO_2_NH_2_}]FeCp (3a)

This compound was prepared according to the general procedure described above, using in this case: (η^5^-C_5_H_4_CHO)FeCp (54 mg, 0.25 mmol) and 4-hydrazinyl-benzenesulphonamide (47 mg, 0.25 mmol). Red solid, yield 56% (53 mg, 0.14 mmol). IR (KBr, cm^−1^): 3407–3302 (νNH/NH_2_); 1603 (νC=N); 1325 (νS-O); 1153 (νS-O). ^1^H NMR (acetone-d_6_): δ 4.17 (s, 5H, C_5_H_5_); 4.34 (st, 2H, C_5_H_4_); 4.62 (st, 2H, C_5_H_4_); 6.28 (s, 2H, NH_2_); 7.11 (d, 2H, *J* = 8.8 Hz, C_6_H_4_); 7.70 (d, 2H, *J* = 8.8 Hz, C_6_H_4_); 7.80 (s, 1H, CH=N); 9.56 (s, 1H, NH). ^13^C NMR (acetone-d_6_): *δ* 66.9 (C_5_H_4_); 68.9 (C_5_H_5_); 69.4 (C_5_H_4_); 80.9 (C_5_H_4ipso_); 111.0 (C_6_H_4_); 127.8 (C_6_H_4_); 133.1 (C_6_H_4_); 139.7 (CH=N); 148.6 (C_6_H_4_). Mass spectrum (*m/z*): 383 [M^+^]. Anal. (%) Calc. for C_17_H_17_N_3_O_2_SFe: C, 53.28; H, 4.47 and N, 10.96; found: C, 53.55; H, 4.36 and N, 10.82.

### 2.3. Synthesis of organometallic-hydrazones [{(η^5^-C_5_H_4_)-C(CH_3_)=N–N(H)-C_6_H_4_-4-SO_2_NH_2_}]MLn(1b, 2b, 3b)

4-Hydrazinyl-benzenesulphonamide (1 eq.) was charged in a two neck round bottomed flask with dried ethanol (10 ml) and a magnetic stir bar. The solution was refluxed under nitrogen atmosphere to obtain a clear solution. To the reaction mixture, acetyl-organometallic precursor (1 eq.) and four drops of HCl were added under stirring condition and the reaction was refluxed for 6 h. After this time, the reaction mixture was filtered and the precipitate was washed with cold hexane (3 × 10 ml) and dried under vacuum for 2 h. The solid obtained was purified using slow diffusion crystallization from THF/hexane (1:5) at −18 °C.

#### 2.3.1. [{(η^5^-C_5_H_4_)-C(CH_3_)=N–N(H)-C_6_H_4_-4-SO_2_NH_2_}]re(CO)_3_ (1b)

This compound was prepared according to the general procedure described above, using in this case: (η^5^-C_5_H_4_COCH_3_)Re(CO)_3_ (60 mg, 0.16 mmol) and 4-hydrazinyl-benzenesulphonamide (30 mg, 0.16 mmol). Brown solid, yield 50% (43 mg, 0.08 mmol). IR (KBr, cm^−1^): 3374–3256 (νNH/NH_2_); 2025 (νRe-CO); 1900 (νRe-CO); 1560 (νC=N); 1328 (νS-O); 1155 (νS-O).^1^H NMR (acetone-d_6_): *δ* 2.02 (s, 3H, CH_3_); 5.74 (t, 2H, *J* = 2.0 Hz, C_5_H_4_); 6.31 (t, 2H, *J* = 2.0 Hz, C_5_H_4_); 6.43 (s, 2H, NH_2_); 7.81 (d, 2H, *J* = 8.8 Hz, C_6_H_4_); 8.13 (d, 2H, *J* = 8.8 Hz, C_6_H_4_); 9.10 (s, 1H, NH). ^13^C NMR (acetone-d_6_): *δ* 21.4 (CH_3_); 85.9 (C_5_H_4_); 86.3 (C_5_H_4_); 103.6 (C_5_H_4ipso_); 128.9 (C_6_H_4_); 130.8 (C_6_H_4_); 138.4 (C_6_H_4_); 140.3 (C=N); 147.8 (C_6_H_4_); 194.9 (Re-CO). Mass spectrum (based on ^187^Re) (*m/z*): 547 [M^+^]; 463 [M^+^ – 3CO]. Anal. (%) Calc. for C_16_H_14_N_3_O_5_SRe: C, 35.16; H, 2.58 and N, 7.69; found: C, 35.26; H, 2.60 and N, 7.68.

#### 2.3.2. [{(η^5^-C_5_H_4_)-C(CH_3_)=N–N(H)-C_6_H_4_-4-SO_2_NH_2_}]Mn(CO)_3_ (2b)

This compound was prepared according to the general procedure described above, using in this case: (η^5^-C_5_H_4_COCH_3_)Mn(CO)_3_ (62 mg, 0.25 mmol) and 4-hydrazinyl-benzenesulphonamide (47 mg, 0.25 mmol). Brown solid, yield 48% (50 mg, 0.12 mmol). IR (KBr, cm^−1^): 3388–3255 (νNH/NH_2_); 2020 (νMn-CO); 1921 (νMn-CO); 1597 (νC=N); 1326 (νS-O); 1154 (νS-O). ^1^H NMR (acetone-d_6_): *δ* 2.10 (s, 3H, CH_3_); 4.92 (s, 2H, C_5_H_4_); 5.43 (t, 2H, C_5_H_4_); 6.90 (s, 2H, NH_2_); 7.85 (d, 2H, *J* = 8.8 Hz, C_6_H_4_); 8.16 (d, 2H, *J* = 8.8 Hz, C_6_H_4_); 9.15 (s, 1H, NH). ^13^C NMR (acetone-d_6_): δ21.4 (CH_3_); 83.0 (C_5_H_4_); 84.1 (C_5_H_4_); 99.3 (C_5_H_4ipso_); 128.0 (C_6_H_4_); 130.0 (C_6_H_4_); 136.5 (C_6_H_4_); 141.4(C=N); 148.2 (C_6_H_4_); Mn–CO (not observed). Mass spectrum (*m/z*): 415 [M^+^]; 331 [M^+^ – 3CO]. Anal. (%) Calc. for C_16_H_14_N_3_O_5_SMn: C, 46.27; H, 3.40 and N, 10.12; found: C, 45.99; H, 3.39 and N, 10.09.

#### 2.3.3. [{(η^5^-C_5_H_4_)-C(CH_3_)=N-N(H)-C_6_H_4_-4-SO_2_NH_2_}]FeCp (3b)

This compound was prepared according to the general procedure described above, using in this case: (η^5^-C_5_H_4_COCH_3_)FeCp (57 mg, 0.25 mmol) and 4-hydrazinyl-benzenesulphonamide (47 mg, 0.25 mmol). Red solid, yield 56% (56 mg, 0.14 mmol). IR (KBr, cm^−1^): 3330–3202 (νNH/NH_2_); 1599 (νC=N); 1330 (νS-O); 1158 (νS-O). ^1^H NMR (acetone-d_6_): *δ* 2.11 (s, 3H, CH_3_); 3.99 (s, 5H, C_5_H_5_); 4.30 (s, 2H, C_5_H_4_); 4.82 (s, 2H, C_5_H_4_); 6.76 (s, 2H, NH_2_); 7.43 (d, 2H, *J* = 8.8 Hz, C_6_H_4_); 7.85 (d, 2H, *J* = 8.8 Hz, C_6_H_4_); 8.77 (s, 1H, NH). ^13^C NMR (acetone-d_6_): δ20.6 (CH_3_); 67.5 (C_5_H_4_); 68.9 (C_5_H_5_); 69.9 (C_5_H_4_); 80.9 (C_5_H_4ipso_); 128.1 (C_6_H_4_); 129.8 (C_6_H_4_); 137.1 (C_6_H_4_); 142.2 (C=N); 155.0 (C_6_H_4_). Mass spectrum (*m/z*): 397 [M^+^]. Anal. (%) Calc. for C_18_H_19_N_3_O_2_SFe: C, 54.42; H, 4.82 and N, 10.58; found: C, 54.46; H, 4.88 and N, 10.55.

### 2.4. CA inhibition studies

An Applied Photophysics stopped-flow instrument has been used for assaying the CA catalysed CO_2_ hydration activity^25^. Phenol red (at a concentration of 0.2 mM) was used as indicator, working at the absorbance maximum of 557 nm, with 20 mM Hepes (pH 7.5) as buffer and 20 mM Na_2_SO_4_ (for maintaining constant the ionic strength), following the initial rates of the CA-catalysed CO_2_ hydration reaction for a period of 10−100 s. The CO_2_ concentrations ranged from 1.7 to 17 mM for the determination of the kinetic parameters and inhibition constants. For each inhibitor, at least six traces of the initial 5−10% of the reaction have been used for determining the initial velocity. The uncatalysed rates were determined in the same manner and subtracted from the total observed rates. Stock solutions of inhibitor (0.1 mM) were prepared in distilled−deionized water, and dilutions up to 0.01 nM were done thereafter with the assay buffer. Inhibitor and enzyme solutions were preincubated together for 6 h at room temperature prior to assay to allow for the formation of the E−I complex. The inhibition constants were obtained by nonlinear least-squares methods using PRISM 3 and the Cheng−Prusoff equation, as reported earlier^26–28^, and represent the mean from at least three different determinations. All CA isoforms were recombinant ones obtained in-house as reported earlier^26^^,^^29^.

## 3. Results and discussion

### 3.1. Synthesis and characterisation of bioorganometallic-hydrazones derivatives from sulphanilamide

**Scheme 1.**
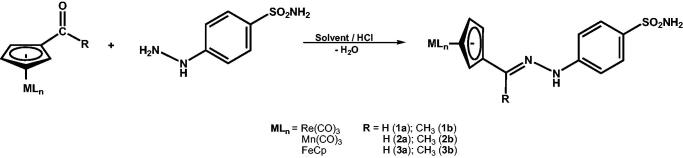
Synthesis of organometallic-hydrazone derivatives **1–3**.

Continuing our interest in organometallic compounds with enzyme inhibitory properties, we decided to prepare such derivatives by using the imine bond formation between 4-hydrazino-benzenesulphonamide and organometallic derivatives incorporating aldehyde or methylketone functionalities. The bio-organometallic-hydrazones of the general formula [{(η^5^-C_5_H_4_)-C(R)=N-N(H)-C_6_H_4_-4-SO_2_NH_2_}]MLn(MLn = Re(CO)_3_, Mn(CO)_3_, FeCp; R=H, CH_3_) were thus prepared by the reaction between formyl or acetyl organometallic precursors and4-hydrazino-benzenesulphonamidein H_2_O (**1a**, **2a**, **3a**) or anhydrous ethanol (**1b**, **2b**, **3b**) in the presence of HCl (Scheme 1). The new compounds were isolated with moderate yields (48–56%) as brown (**1a–b**, **2a-b**) or red (**3a–b**) solids after crystallization from acetone/hexane or THF/hexane, and exhibited good solubility in most polar organic solvents.

All compounds were characterised by electron impact (EI) mass spectrometry, elemental analysis, infra-red and NMR spectroscopies (Supporting Information). A strong molecular ion was shown in the mass spectrum of each organometallic-hydrazone, in addition to the detection of notable successive losses of CO ligands for the cyrhetrenyl (**1a–b**) and cymantrenyl (**2a–b**) derivatives (Supplementary Figure S1†). The elemental analysis data determined for all compounds is in agreement with their proposed formulas.

The FT-IR spectra (KBr disk) of all compounds showed the expected absorption bands for the νN–H, νC=N and νSO_2_ stretches in the ranges of 3407–3202 cm^−1^, 1603–1560 cm^−1^ and 1330–1147 cm^−1^, respectively (Supplementary Figure S2†). Similar frequency values have been reported for others organic^30^^,^^31^ and organometallic-hydrazones^32–34^. In addition, IR analysis of compounds **1a–b** and **2a–b** revealed the presence of two intense bands in the region of 2025–1900 cm^−1^, that are characteristic of the asymmetric (ν_as_) and symmetric (ν_s_) stretchings of the terminal CO ligands of cyrhetrene and cymantrene derivatives^35–37^ (Supplementary Figure S3†).

It is well-known that hydrazones may adopt two different forms (*E*- or *Z*-)^38^ in the solid state and also in solution. ^1^H and ^13^C{1H} NMR spectra of all compounds showed only one set of resonances, thus suggesting that only one of the two isomers was present in acetone-d_6_ solution at room temperature. On this regard, ^1^H-NMR spectra of **1a**, **2a** and **3a** showed exhibited a sharp singlet was observed in the ranges of 7.80–7.64 ppm, and it was assigned to the iminic proton. In addition, a signal due to the methyl protons of the –C(CH_3_)=N–fragment was also observed at approximately *δ* 2.1 for compounds **1b**, **2b** and **3b**. These results are in agreement with the values reported for organic^39^ and organometallic analogues^40^^,^^41^. In addition, the resonances observed between 8.16 and 7.11 ppm were assigned to the hydrogen atoms of the C_6_H_4_ ring. As per literature reports, the broad singlet observed at 6.90–6.28 ppm was assigned to the hydrogen nuclei of the SO_2_NH_2_ group^42^^,^^43^. Moreover, ^1^H NMR spectra for **1a**-**b** and **2a**-**b** showed sets of resonances in the region of 6.31–4.92 ppm, which are ascribed to the protons of the cyrhetrenyl and cymantrenyl moieties^44^^,^^45^. On this regard, the ferrocenyl derivatives **3a–b** exhibited resonances around *δ* 4.82–4.30 due to the non-equivalent alpha and beta protons containing in the substituted Cp ring and a singlet in the region of 4.17–3.99 ppm, which was assigned to the proton resonances of the unsubstituted cyclopentadienyl group^46^^,^^47^. The presence of the NH group registered as a broad singlet in the range of 9.9–8.7 ppm. Similar *δ* have been reported for other organometallic to sylhydrazones^48^. It is important to note that the chemical shifts of the NH resonance showed a clear dependence on the presence of the organometallic moiety bound to the iminic entity. In fact, the downfield shift observed for the cyrhetrenyl (**1a–b**) and cymantrenyl (**2a–b**) tosyl HYDs (Δ*δ* ∼ 0.30) compared with ferrocenyl analogues (**3a–b**) can be related to the electron-withdrawing properties of the (η^5^-C_5_H_4_)M(CO)_3_ moieties^49^, which produce a deshielding of the NH resonance, thus, suggesting that the nature of the organometallic framework modifies the degree of electronic delocalisation on the –C(R) = N–NH– unit. We have found similar results for ferrocenyl and cyrhetrenyl 1,3,4-thiadiazoles^50^ and thiosemicarbazones^51^.

The ^13^C NMR data are also in agreement with the proposed structures, that is, all compounds showed the carbon nuclei of the organometallic fragments, C=N bridge and phenyl moiety. As expected, the resonances for the carbon atoms of the CH_3_ and C_6_H_4_ groups were observed at *δ* 21 and 155–110 and did not show any noticeable differences from those reported for the organic^52^ and organometallic analogues^48^^,^^53^. The most important feature of the ^13^C NMR spectra is the presence of a low field resonance (148–131 ppm), which was assigned to the iminyl carbon [C=N].

The carbon chemical shifts of this group for **1a**, **2a** and **3a** also showed a clear dependence on the electronic properties of the organometallic moiety attached to it. The upfield shift observed for the cyrhetrenyl (**1a**) and cymantrenyl (**2a**) tosyl HYDs (∼132 ppm) compared with the ferrocenic analogue (**3a**) (140 ppm) can also be related to the opposite electronic effects of these organometallic moieties. This proposal is in agreement with the trend observed in the resonance of the NH proton mentioned above. We previously reported similar results for Schiff bases^54^, thiosemicarbazones^51^ and hydrazones^55^ containing ferrocenyl and cyrhetrenyl moieties. This phenomenon is not observed in compounds **1b**, **2b** and **3b**, in which the hydrazone fragment possess a methyl group attached to the C=N entity. In these cases, we believe that the inductive effect of the CH_3_ substituent could better stabilise any charge generated in the hydrazone entity; therefore, no difference in the iminyl carbon resonance would be evidenced. It is important to mention that all assignments were confirmed by ^1^H ^13^C NMRHSQC (Supplementary Figure S4†).

### 3.2. CA inhibition studies

The obtained sulphonamides (**1–3)a,b** were investigated for their CA inhibitory properties by using a stopped-flow CO_2_ hydrase assay^25^ and four human CA isoforms (hCA I, II, IX and XII) known to be drug targets^1–5^ (Table 1).

**Table 1.** hCA I, II, IX and XII inhibition data with compounds **(1–3)a,b** and acetazolamide (AAZ) as standard drug, by a stopped-flow CO2 hydrase assay [25].


K_I_ (nM)^a^				
	hCA I	hCA II	hCA IX	hCA XII
**1a**	7.5	22.4	579.8	24.4
**2a**	5.9	8.2	2.1	3.1
**3a**	13.2	1.7	49.9	4.9
**1b**	3.7	9.7	25.0	3.8
**2b**	268.6	11.2	221.3	3.3
**3b**	3.6	9.3	41.9	1.9
**AAZ**	250	12	25.8	5.7

^a^ Mean from three different assays, by a stopped flow technique (errors were in the range of ± 5–10% of the reported values).

The new organometallic derivatives exhibited effective inhibition of the cytosolic isoforms hCA I and II, with inhibition constants in the range of 1.7–22.4 nM, except for **2b** which was a moderate-weak hCA I inhibitor (*K*_I_ of 286.6 nM; Table 1). Thus, apart this outlier compound **2b**, the nature of the metal ion, the presence/absence of CO moieties or the aldehyde/ketone nature of the starting materials did not influence significantly the CA inhibitory properties. The ferrocenyl derivatives **3a** and **3b** were slightly more effective as CAIs compared to the structurally similar derivatives, but the differences were not impressive. Against hCA IX, a transmembrane, tumour-associated isoform, only **2a** was a low nanomolar inhibitor, and **1b** and **3b** had K_I_ values <50 nM. The remaining derivatives showed medium potency inhibitory action, with inhibition constants in the range of 221.3–579.8 nM. However, the second transmembrane, tumour-associated isoform hCA XII was effectively inhibited by all new compounds reported here, with *K*_I_ values in the range of 1.9–24.4 nM. The structure**–**activity relationship (SAR) is thus quite flat, with a very modest range of variation in the inhibition constant values. Anyhow, the best CAI was **3b** with an inhibition constant of 1.9 nM (ferrocenyl derivative) and the least effective one was the rhenium carbonyl derivative **1a** with a *K*_I_ of 24.4 nM.

## 4. Conclusion

A small number of organometallic benzenesulphonamide derivatives were obtained by reaction of aldehydes/ketones incorporating ferrocenyl or rhenium/manganese tricarbonyl moieties with 4-hydrazino-benzenesulphonamide. The new compounds were investigated as inhibitors of four pharmacologically relevant human CA isoforms, hCA I, II, IX and XII. The cytosolic isoforms hCA I and II were effectively inhibited by almost all derivatives with inhibition constants in the range of 1.7–22.4 nM. The same effect was observed for the tumour-associated transmembrane isoform hCA XII, for which K_I_s in the range of 1.9–24.4 nM were measured. hCA IX was on the other hand less sensitive to inhibition with these compounds, with only one derivative showing low nanomolar inhibitory action. The presence of metallo-organic or metallo-carbonyl moieties in the molecules of these CAIs makes them amenable for interesting pharmacologic applications, for example for compounds with CO donating properties.

## Supplementary Material

Supplemental Material
