# Supplementary material for: Bioorganometallic derivatives of 4-hydrazino-benzenesulphonamide as carbonic anhydrase inhibitors: synthesis, characterisation and biological evaluation
Source: J Enzyme Inhib Med Chem. 2020 Feb 10;35(1):622–8. doi: 10.1080/14756366.2020.1724995 (PMC7034112; doi:10.1080/14756366.2020.1724995)

SUPPORTING INFORMATION FOR

Bioorganometallic derivatives of 4-hydrazino-benzenesulfonamide as carbonic anhydrase inhibitors: Synthesis, characterization and biological evaluation

Jeremie Brichet [a], Rodrigo Arancibia [a,*], Emanuela Berrino [b], Claudiu T. Supuran [b,*]

[a] Laboratorio de Química Inorgánica y Organometálica, Departamento de Química Analítica e Inorgánica, Facultad de Ciencias Químicas, Universidad de Concepción, Concepción, Chile.

[b] Dipartimento Neurofarba, Sezione di Scienze Farmaceutiche, Università degli Studi di Firenze, Firenze, Italy.

*Corresponding authors: E-mail: rarancibia@udec.cl (Rodrigo Arancibia); E-mail: claudiu.supuran@unifi.it; Tel/Fax: +39-055-4573729 (Claudiu T. Supuran)

Supporting information contents:

1.- Supplementary Figures

Figure S1. Electron impact mass spectrum of **1a**.

Figure S2. FT-IR spectrum (KBr disk) of **3b**.

Figure S3. FT-IR spectrum (KBr disk) of **1a**.

Figure S3. [^{1}H-^{13}C]-HSQC spectrum of ligand **2a** in acetone-d_6 at 298 K.

Figure S1. Electron impact mass spectrum of **1a**.

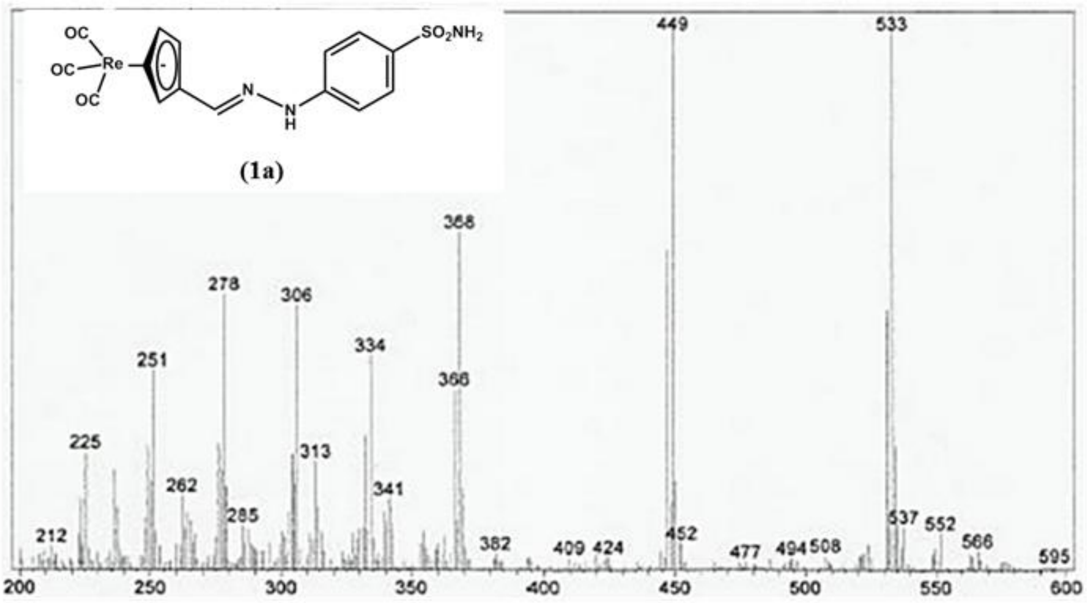

Figure S2. FT-IR spectrum (KBr disk) of **3b**.

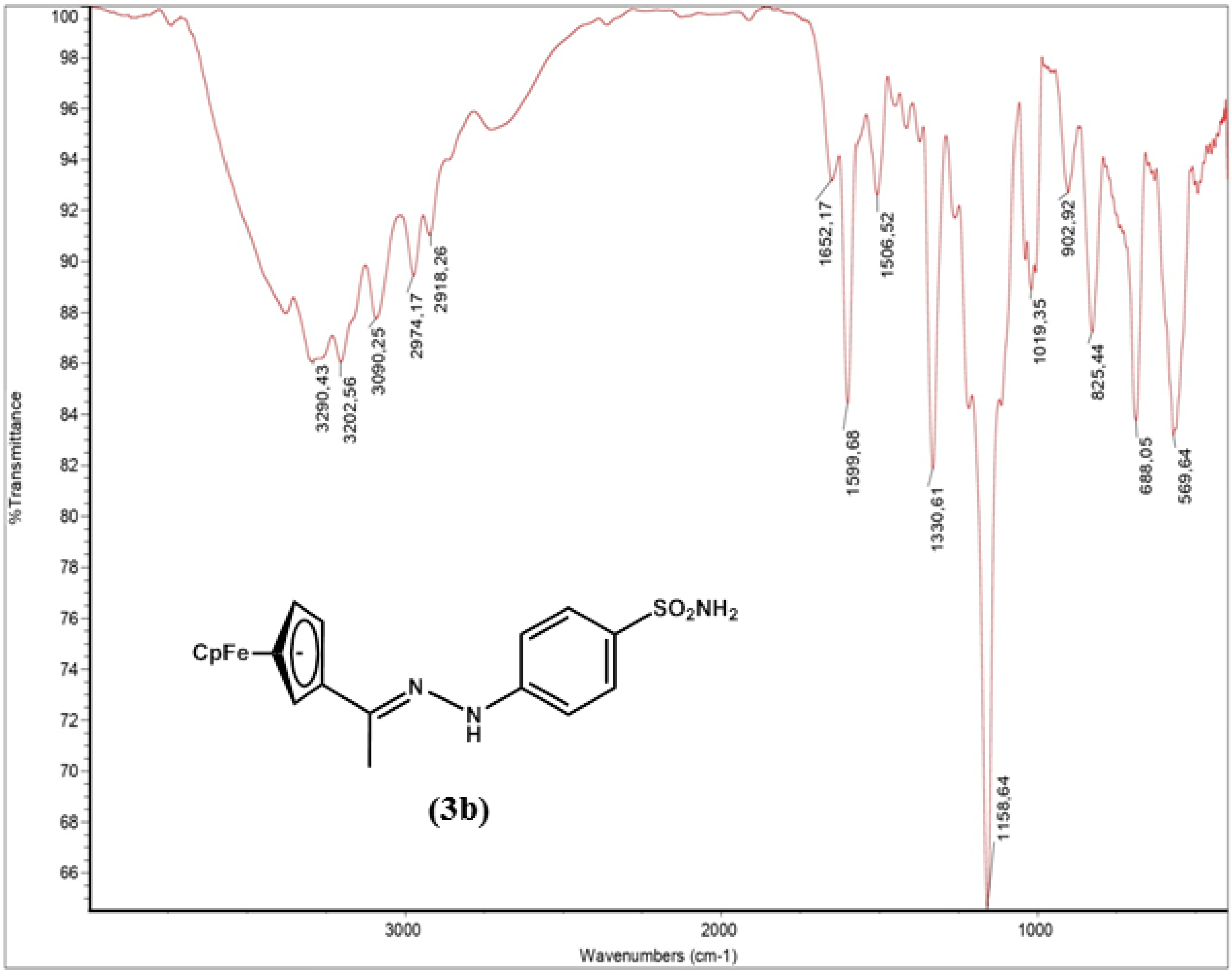

Figure S3. FT-IR spectrum (KBr disk) of **1a**.

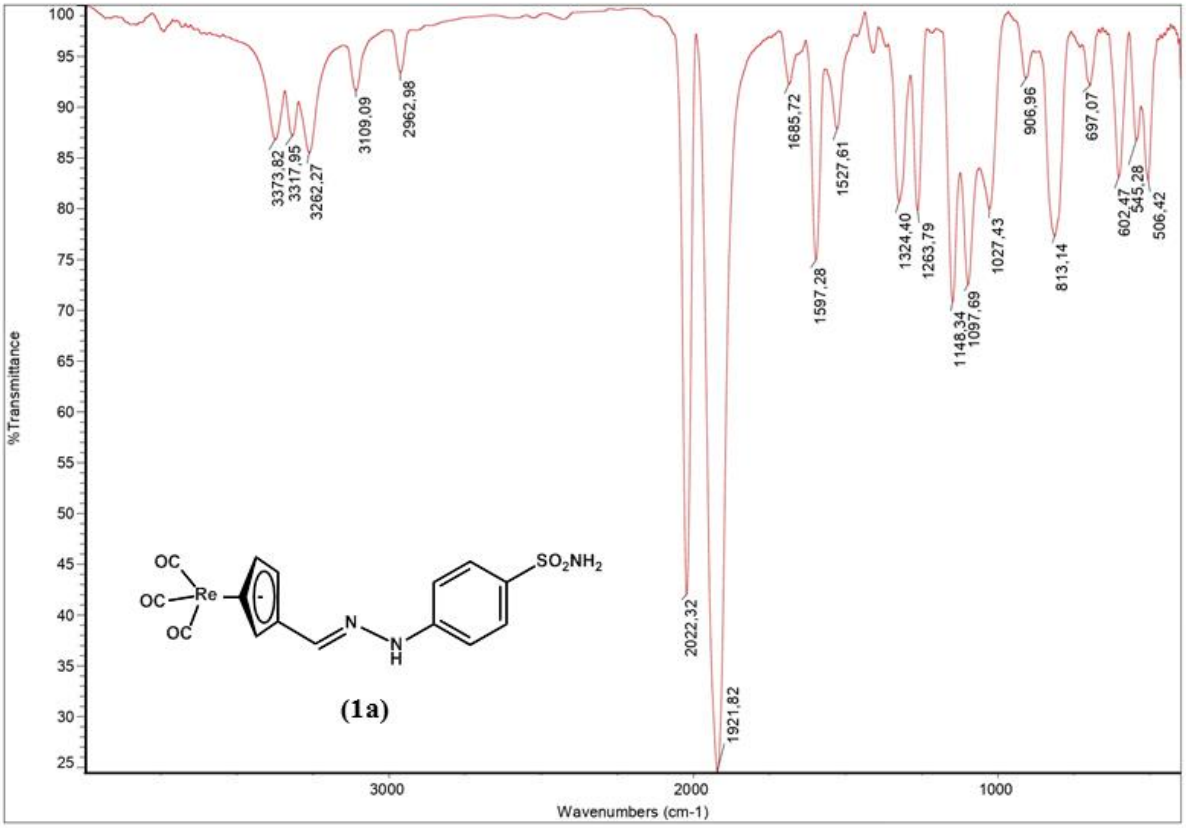

Figure S4. [^{1}H-^{13}C]-HSQC spectrum of **2a** in acetone-d^6 at 298 K.

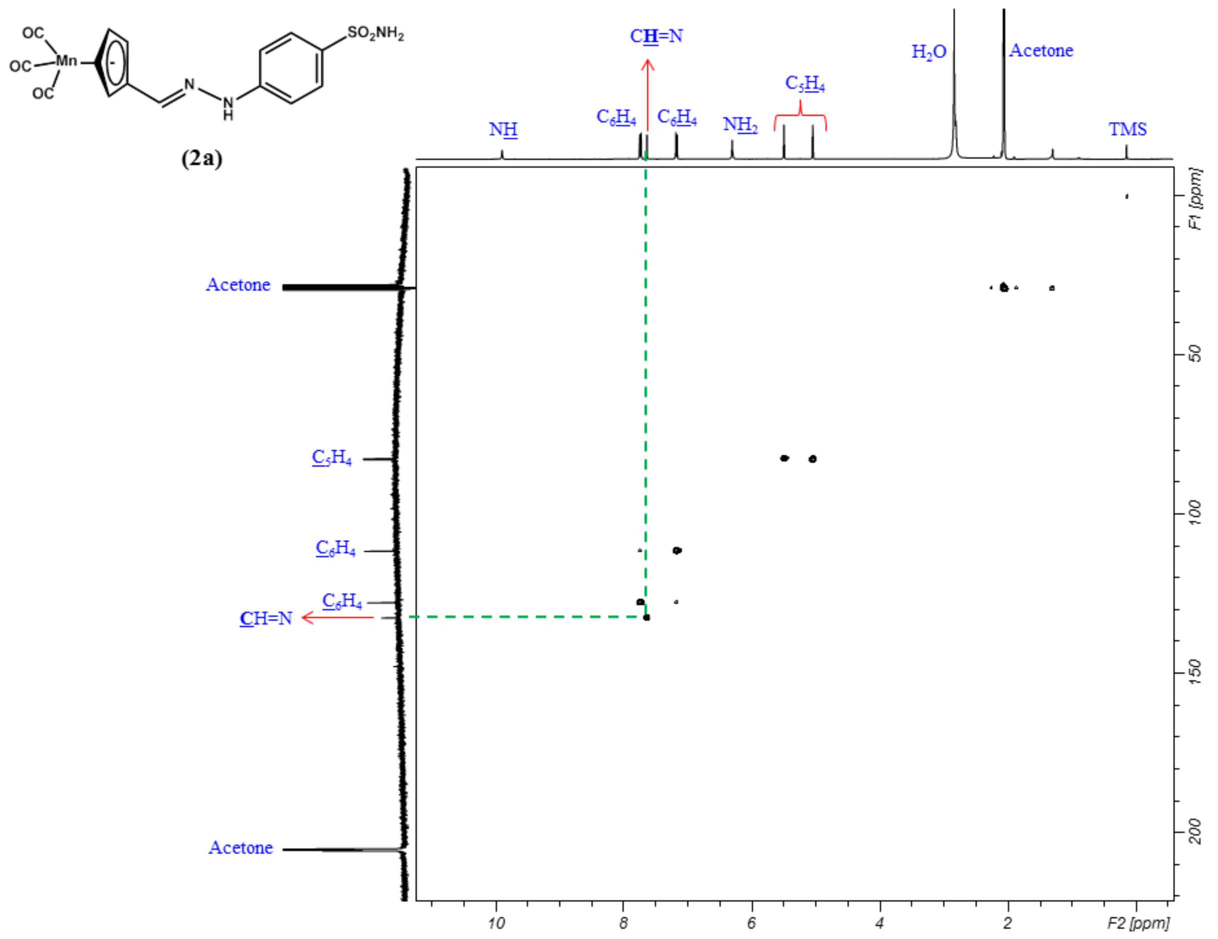

Supplement: Supplemental Material [file IENZ_A_1724995_SM4433.pdf]
